# DSM-5: the debate continues

**DOI:** 10.1186/2040-2392-4-11

**Published:** 2013-05-15

**Authors:** Joseph D Buxbaum, Simon Baron-Cohen

**Affiliations:** 1 Seaver Autism Center for Research and Treatment, Departments of Psychiatry, Neuroscience and Genetics and Genomic Sciences, Friedman Brain Institute, Icahn School of Medicine at Mount Sinai, New York NY 10029, USA; 2 Autism Research Centre, Cambridge University, Cambridge CB2 8AH, UK

## Abstract

We are fortunate to have invited commentaries from the laboratories of Dr Cathy Lord and Dr Fred Volkmar offering their perspectives on the new Diagnostic and Statistical Manual of Mental Disorders (DSM)-5 criteria for the autism spectrum. Both commentaries note how DSM-5 collapses the earlier diagnostic categories of the pervasive developmental disorders into a single category of autism spectrum disorder. In addition, DSM-5 collapses social and communication domains into a single combined domain. The commentaries go on to discuss the positive aspects of these changes and raise some areas of potential concern. We support the evidence-based changes to autism diagnosis found in DSM-5, and look forward to further studies on the autism phenotype as this has implications for diagnosis and treatment. As our mechanistic understanding of autism improves, diagnoses based on behavioral parameters will continue to provide opportunities for interventions targeting the behaviors, while etiological diagnoses will provide opportunities for interventions tailored to etiology.

## 

We are fortunate to have invited commentaries from the laboratories of Dr Cathy Lord and Dr Fred Volkmar offering their perspectives on the new Diagnostic and Statistical Manual of Mental Disorders (DSM)-5 criteria for the autism spectrum.

Both Lord and Volkmar are world-leaders in autism and in the autism phenotype, and both have been very involved in the DSM: Volkmar was the primary author of the DSM-IV autism and pervasive developmental disorders section, and Lord has been equally active in the Neurodevelopmental Disorders Workgroup of DSM-5. As such, there are none more qualified to comment on what has been potentially gained or lost in the transition from the fourth edition to the fifth edition of this bible of psychiatric classification and diagnosis.

DSM-5 presents an interesting new approach to the diagnosis of the autism spectrum. On the one hand, as both commentaries highlight, DSM-5 collapses the show $132#?>earlier diagnostic categories of pervasive developmental disorders into a single category, termed autism spectrum disorder (ASD), and further collapses the communication and social domains into a single social-communication domain.

At the same time, a subtype of pervasive developmental disorders that is likely to be distinct and map to unique and identifiable etiology factors (i.e., Rett syndrome) has been moved out of the ASD category. Overall, these changes have the effect of a greater focus on the behavioral manifestations of ASD, encompassing both domains and severity, and a move away from etiological considerations.

The commentary by Lord’s group is broadly positive about DSM-5 for three reasons: DSM-5 has merged five subgroups with low inter-rater reliability into a single group with high inter-rater reliability; DSM-5 has reduced the autistic spectrum from three factors down to two, in recognition that social and communication skills are inextricably intertwined; and DSM-5 has introduced a severity scale in recognition of the diversity of the spectrum.

The commentary by Volkmar’s group raises some concern about DSM-5 for three reasons: DSM-5 will make it difficult for ongoing longitudinal research studies to compare like with like; there is some evidence (reviewed by them) that some high-functioning individuals will no longer meet diagnostic criteria for ASD, and will thereby become ineligible for services and treatment; and the removal of the subgroup of Asperger syndrome is too extreme a move, when its reliability could have been improved by some tweaking, retaining its clinical value, rather than by deleting it entirely. What impact removing Asperger syndrome from DSM-5 will have on individuals who now feel this as part of their identity remains to be seen.

The Volkmar commentary goes on to argue that we do not know what the broader impact of the DSM-5 changes will be. If DSM-5 does lead to a percentage of people on the autism spectrum no longer meeting criteria for a diagnosis and thereby losing services, this is very serious indeed. We know that many high-functioning individuals with superficially very good social skills may nevertheless struggle psychologically – that outward behavior can mask inner suffering – and that comorbid depression is common in this group. Without appropriate services, such individuals may acquire even greater risk of secondary depression, with all that this implies in relation to suicidality risk. It will be important for future clinical research to monitor the impact of DSM-5 carefully, to see whether these fears are well-founded or unfounded.

More subtle changes in DSM-5 include the inclusion of stereotyped language into the repetitive and restrictive behaviors domain, and the addition of sensory issues into the definition. The former change is supported by empirical data. The latter change is more exploratory, recognizing that sensory over-responsivity or under-responsivity is frequently reported in ASD. It will be interesting to see how sensory processing disorders unfold when clinicians and researchers adopt standard instruments to report such sensory processing changes in ASD.

Other changes in DSM-5 may impact the diagnosis of ASD. Most importantly, the addition of a new category of social-communication disorder may lead to diagnostic changes that are not currently predictable. This latter point is perhaps the one that has caused the greatest discussion. We simply cannot predict how changing diagnostic criteria impact the real world. The DSM-5 committees are to be commended for the work in trying to assess impact, but there are far too many factors and affected parties to be able to make sanguine predictions. For example, a township or insurance company seeking to reduce its financial obligation could perhaps use changing diagnostic criteria to argue that certain individuals no longer qualify for specific services. If this occurs, for the families in question the impact will be profound, and the ensuing delays in services very worrying.

In an ideal world, where appropriate and feasible, behavioral diagnosis of ASD would be coupled with an etiological diagnosis. Such a dual approach would then leverage evidence-based treatment (whether pharmacological or psychological) that takes advantage of interventions tailored to the individual behavioral expression and etiology. For example, in a child with Rett syndrome, ASD deficits might be addressed through behavioral interventions, while neurobiological alterations that underlie the behavioral changes might be addressed by novel therapeutics developed to target specific changes caused by the loss of a functional copy of the *MECP2* gene. A parallel phenotype/etiology approach could be applied to other high-risk mutations in ASD, such as mutations in *FMR1*, *MBD5*, *SHANK3*, *TSC1* or *TSC2*, and *UBE3A*, copy number variation at 15q11-13, 16p11, 22q11, and 22q13, and smaller or larger chromosomal disorders.

We thank the Lord and Volkmar groups for contributing to this important debate, and look forward to publishing more research evaluating these diagnostic changes. The decision to include these commentaries also reflects the editorial vision of *Molecular Autism*, which is to understand autism at multiple levels, whether molecular, neural, cognitive or behavioral. To reflect this vision, *Molecular Autism* now has a strapline: *Brain, Cognition, and Behavior*. We encourage submissions of articles for publication reporting high-quality autism research at all of these levels.

## Abbreviations

ASD: Autism spectrum disorder; DSM: Diagnostic and statistical manual.

